# Exploring the peer status prototypes: A large‐scale latent profile analysis on high‐school students from four European countries

**DOI:** 10.1111/sjop.12863

**Published:** 2022-08-08

**Authors:** Marco Marinucci, Luca Pancani, Paolo Riva

**Affiliations:** ^1^ University of Milano‐Bicocca Milano Italy

**Keywords:** Peer status, sociometric status, interpersonal processes, latent profile analysis, peer nomination

## Abstract

Peer status – the regard other group members have of an individual – is fundamental for youth development. Different research traditions developed independent theoretical frameworks conceiving the dimensions underlying social status, and this led to identifying a variety of peer status prototypes. In this work, we explored whether a classification based on the four dimensions of popularity, aggression, dislike, and victimization could integrate the scattered peer status profiles found in the different traditions. A latent profile analysis on 16,224 European students identified the peer status prototypes of popular, bullies, disliked, victims, and average students. Both the peer‐ and self‐reported correlates supported that the five profiles accounted for the large variety of the students' profiles in the literature. These findings suggest that the adoption of a multidimensional approach supported by advanced statistical procedures could identify students' peer status profiles more effectively, replacing classifications based on cutoffs, and leading to a unified students' classification.

## INTRODUCTION

Decades of research in psychology have shown that peer relationships are crucial for children's development (Hartup, 1970). Poor peer relationships and psychological problems are reciprocally intertwined from early childhood to adolescence (Hay, Payne & Chadwick, 2004; Ladd & Troop‐Gordon, 2003). Difficulties in peer relationships have been associated with disruptive behaviors (Pedersen, Vitaro, Barker & Borge, 2007), internalizing problems (Reijntjes, Kamphuis, Prinzie & Telch, 2010), psychosocial difficulties and psychotic experiences (El Bouhaddani, van Domburgh, Schaefer, Doreleijers & Veling, 2018), and poor school achievement (Flook, Repetti & Ullman, 2005), ending up in a later educational under‐achievement and unemployment (Woodward & Fergusson, 2000). Given the broad spectrum of adverse outcomes associated with problematic peer relationships – and the positive outcomes associated with supportive ones (Anderson, John, Keltner & Kring, 2001; Anderson, Kraus, Galinsky & Keltner 2012) – the assessment of the students' social status has received considerable attention from researchers measuring the *peer status*. The peer status describes the relationship between an individual and the group, qualifying the consideration received from other members along emotional (e.g., being liked or disliked) and reputational (e.g., visibility and impact) parameters (Ilmarinen, Vainikainen, Verkasalo & Lönnqvist, 2019).

The main scientific approaches to the students' peer status are the *sociometric, sociological*, and *evolutionary* research traditions, which identified different sets of students' prototypes. Among them, some conceptually overlapped, some were specific to the singular approaches, and some others were further differentiated in subtypes by subsequent classifications. In this work, we aimed at applying an advanced clustering technique (i.e., latent profile analysis) to a large‐scale and international dataset of 16,224 European students to explore whether a more comprehensive theoretical framework could account for the existing peer status prototypes.

### The sociometric research

The traditional sociometric research focused on acceptance and rejection – measured by asking students to nominate their classmates they most liked and disliked (Gifford‐Smith & Brownell, 2003) – to derive the theoretical dimensions of the sociometric classification. The *social preference*, measuring likability, was computed by subtracting the number of disliking from the liking nominations, whereas the *social impact*, measuring visibility and influence, was computed by summing the two (Newcomb & Bukowski, 1983).

In the traditional sociometric classification, the researchers used cutoffs on the dimensions of preference and impact and on the indices of acceptance and rejection to identify five categories of students (Coie, Dodge & Coppotelli, 1982). The popular students – as called in the early classification (Coie *et al*., 1982) – were those highly preferred by others, presenting high scores on the social preference dimension that indicated a high likeability among their peers. Given that this profile was defined only by liking nominations and not popularity, a more recent classification proposed to refer to them as *well*‐*liked* students (Cillessen & Marks, 2011). The well‐liked students possessed high prestige in their group, and they were depicted as prosocial, leaders, with low aggressive behaviors (Newcomb, Bukowski & Pattee, 1993; Rubin, Bukowski & Parker, 2006). The *rejected* students were those highly disliked by others, showing low social preference scores. They showed high levels of aggression, conduct and attentional problems, and poor school achievements (Ollendick, Weist, Borden & Greene, 1992), as well as high levels of social withdrawal, depression, and anxiety (Cillessen, Van IJzendoorn, van Lieshout & Hartup, 1992; Newcomb *et al*., 1993). Many studies on the rejected students distinguished between the *aggressive* and *withdrawn* subtypes (e.g., Inderbitzen, Walters & Bukowski, 1997). Cillessen and colleagues (Cillessen *et al*., 1992) found that aggressive‐rejected children were disruptive, impulsive, hypersensitive, dishonest, and noncooperative. By contrast, withdrawn‐rejected students were more shy and friendless, with higher feelings of loneliness, depression, social anxiety, negative self‐perceptions, and lower self‐esteem (Boivin, Poulin & Vitaro, 1994; Boivin & Bégin, 1989; Cillessen *et al*., 1992; Inderbitzen *et al*., 1997). Besides the more common aggressive‐withdrawn differentiation, other scholars focused on the interaction between social exclusion (e.g., rejection) and victimization. Schuster (1999, 2001) further differentiated the sociometric rejected students into the rejected‐only and the victimized‐rejected, respectively clustering students left apart from others and those left apart and victimized by their peers. Those who received high numbers of both liking and disliking nominations were classified as *controversial* students. They were described as both possessing leadership and sociable interpersonal skills (Lansu & Cillessen, 2012) and arrogant, aggressive, and snobbish (Carboni & Casciaro, 2013; Newcomb *et al*., 1993). Consistently with the combination of strong influence and aggressivity (Lease, Musgrove & Axelrod, 2002), the controversial students were often identified as bullies and bully‐victims (e.g., Boulton & Smith, 1994; Farmer, Petrin, Robertson *et al*., 2010), holding a dominant position in the classroom social structure (Olweus, 2003). Lastly, the *neglected* students received a small number of nominations, indicating their low relevance to their classmates, as if they were forgotten or invisible. This sociometric category showed poor temporal stability, behavioral discriminability, and representativeness – given the low number of students usually categorized in these profiles (Cillessen *et al*., 2000; Frederickson & Furnham, 1998; Gifford‐Smith & Brownell, 2003; Hill & Merrell, 2004; Ollendick, Greene, Francis & Baum, 1991; Terry & Coie, 1991). Moreover, researchers failed to identify the discriminant characteristics of these students (Rubin *et al*., 2006). Thus, the neglected students appeared to be more similar to the average students than different from them (Brown, 2015), leading some authors to doubt the meaningfulness of this category (Rubin, Hymel, Lemare & Rowden, 1989).

### The sociological research

The sociological research addressed the analysis of *popularity* as the main indicator of social status. In the sociological research, popularity indicates power, prestige, and visibility – hence, overlapping with the sociometric dimension of social impact – and it is conceived as the primary indicator of the students' social status (Cillessen & Marks, 2011).

Focusing on the children's social construction of who and what makes someone popular by asking students to nominate the most and least popular ones, sociological researchers identified a group of students referring to them as the *popular clique* (Adler & Adler, 1998). Such a heterogeneous group of students at the top of the social hierarchy showed strong influence among their peers. They were described as engaging in prestigious activities and possessing the “expressive equipment” of social prestige (e.g., clothing; Adler, Kless & Adler, 1992, p. 173). They also showed shrewd interpersonal skills often used to manipulate others and maintain their position at the top of the hierarchies (Adler & Adler, 1998).

From this early sociological contribution, the sociometric and developmental researchers identified two different subtypes of *perceived popular* students. Rodkin, Farmer, Pearl and Van Acker (2000) identified the *model*, perceived as prosocial, not shy, and not aggressive, and the *tough*, perceived as aggressively and antisocially dominant. Similarly, de Bruyn and Cillessen (2006) distinguished between the *populistic* – aggressive, stuck up, and bullies – and the *prosocial* populars – affiliative, helpful, and prosocial.

### The evolutionary research

The evolutionary tradition mainly focused on the different strategies that students can display to achieve social influence and power in the peer‐group structure. Hawley (1999) highlighted the control of material (e.g., toys) and social (e.g., consensus) resources as indices of influential status in the peer group. She differentiated two main profiles of late middle‐school students: the *bullies* relying on coercive strategies (e.g., interrupting, fighting), and the *leaders* utilizing prosocial ones (e.g., inviting, requesting). Whereas leaders are favored and liked by their peers, bullies are not, and they may experience higher rates of social exclusion and struggle more in maintaining amicable relationships. Both the bullies' and the leaders' strategies are effective in achieving an influential status in the group, at the expense of the low‐dominant, undifferentiated others.

Although the research did not extensively investigate the psychological correlates of the evolutionary profiles of the leaders and bullies, Lease *et al*. (2002) pointed out that the leaders and the sociometric well‐liked students – as well as the prosocial subtypes of the sociometric popular – may reasonably identify the same prototype of students, given that the core characteristics they share tap into social influence, leadership and prosocial qualities. Similarly, the bullies, the antisocial subtypes of the popular, and the sociometric controversial may overlap, given that all of them showed simultaneously aggressive, high status, and bossy characteristics, while also being disliked.

### The need to adopt new analytical approaches to explore the students' social status

Considering the three reviewed approaches, the current knowledge of the students' peer status appears confusing. The different research traditions independently identified different dimensions of the students' social status. This led the different research traditions to identify similar sets of prototypes that apparently overlapped each other. The well‐liked sociometric students showed the same social, psychological, and behavioral characteristics as the prosocial (model) popular, and the leaders; the controversial showed the same as the populistic (tough) popular and the bullies. Therefore, the literature seemed to provide converging evidence regarding the peer status prototypes, although the various theoretical dimensions considered and the diverse terminology adopted for the profiles' names made the literature fuzzy. To make this scenario even more confusing is the fact that the specific subtypes of the rejected‐only and victimized‐rejected students identified by Schuster (1999, 2001) or the aggressive‐ and withdrawn‐rejected students (Inderbitzen *et al*., 1997) were rarely represented or accounted for in none of the main theoretical frameworks investigating the social status (see Table 1).

**Table 1:** Theoretical backgrounds of the peer‐status prototypes

	Overlapping profiles					
Coie *et al*., 1982	Well‐liked Popular	Controversial	Rejected		Neglected	Average
Rodkin *et al*., 2000; de Bruyn & Cillessen, 2006	Model/Prosocial Popular	Tough/Populistic Popular				
Hawley, 1999	Leader	Bully				
Schuster, 1999			Rejected‐only	Rejected‐victimized		
Inderbitzen *et al*., 1997			Aggressive‐Rejected	Withdrawn‐Rejected		
Gifford‐Smith & Brownell, 2003; Hill & Merrell, 2004; Rubin *et al*., 1989, 2006; Brown, 2015					Average	

In this work, we tried to explore a new analytical approach that could possibly integrate in a heuristic fashion the contributions from all these scientific approaches. Empirically, this would result in the identification of a reduced set of peer status profiles accounting for the larger constellation of prototypes. Such classification could also explain the difference in the subtypes of low‐status students (aggressive/withdrawn; rejected‐only/rejected‐victimized), as we would consider the role of aggressiveness in peer dynamics not only as a way to the top of the social hierarchy – as so far done by previous literature (Hawley, 1999; Rodkin *et al*., 2000), but also as a factor distinguishing between different low‐status profiles.

### The present study

In the present study, we aimed at identifying a multidimensional peer status classification integrating the profiles identified in different research traditions. We did so using a latent profile analysis to derive the peer status profiles and relying on a large‐scale dataset of 16,224 European students. Moreover, we compared the social, behavioral, and psychological self‐reported correlates of the identified profiles with the peer‐reported nomination and the previous literature on the profiles' characteristics, further assessing the face validity of the resulting profiles.

We considered simultaneously the index of popularity, aggression, dislike, and victimization as fundamental dimensions of the peer status classification. Indeed, we expected that on one hand, the simultaneous consideration of popularity and aggression would be able to discriminate the well‐liked, the prosocial and model popular, and the leader (i.e., the prosocial subtype of popular students) from the controversial students, the tough and populistic popular, and the bullies (i.e., the aggressive subtype of populars). On the other hand, the concurrent consideration of the dimensions of social exclusion and victimization would discriminate between the rejected‐only and rejected‐victimized students. In doing so, we decided to adopt an explorative approach to the data. Furthermore, the inclusion of the two indices of exclusion, victimization, and aggression should distinguish between the aggressive and nonaggressive (withdrawn) subtypes of rejected students (Inderbitzen *et al*., 1997). We computed the peer status classification by adopting a latent profile analysis (LPA; Nylund‐Gibson & Choi, 2018). The latent profile analysis can be a useful statistical method to identify the students' profiles relying on statistical parameters rather than on cutoffs – as traditionally done in the sociometric approach (Coie *et al*., 1982). LPA allows identifying subgroups of individuals (i.e., classes) that are similar in a set of characteristics. Compared to the use of the cutoffs – which would generate “essentially artificial” profiles (Cillessen & Bukowski, 2000), LPA has two main advantages. First, the number of classes that are selected is based on statistical indices that are more precise and sophisticated than the classical methods based on the distance between pairs of cases or between cases and clusters' centroids. Second, for each individual, LPA computes the probability to be a member of each class, taking into account errors in the estimation and providing indices of the precision of the categorization (i.e., posterior probabilities). Using LPA, Hubbard, Smith and Rubin (2013) showed that the traditional sociometric classification did not correspond to the one that emerged from a latent profile analysis, highlighting how the traditional methodology may not be sensitive enough to represent the complexity of the peer relations. The peer status classification of the present work resulting from the LPA was grounded on *statistically* significant patterns of information carried by the observed data (i.e., grouping the students according to the likelihood of their membership to a profile), rather than on an a priori computation via the cutoffs. In doing so, we based the classification on a sample of 16,224 high‐school students in four European countries (the Netherlands, England, Germany, and Sweden). The large sample collected in different countries allowed us to provide high‐powered statistical analyses, and to extend the generalizability of the findings of the students' peer status profiles at a European level.

Lastly, we considered relevant social (i.e., intergroup social connections and dating experiences), psychological (i.e., perceived victimization, self‐esteem, and violent masculinity attitude), behavioral (i.e., behavioral problems in school), and well‐being (i.e., general health and satisfaction with life) self‐reported correlates of the identified peer status profiles to check their construct validity. We considered the self‐reported correlates that the literature showed to be relevant in discriminating between the different profiles (e.g., de Bruyn & Cillessen, 2006; Gifford‐Smith & Brownell, 2003).

In detail, we expected that the most popular profiles would show higher social integration and attractiveness (operationalized by intergroup social connections and dating experiences) and well‐being (self‐esteem, satisfaction with life, and general health) than the least popular and most rejected ones. We also expected that the behavioral problems in school and violent masculinity attitudes would distinguish the prosocial popular from the aggressive ones, as well as the aggressive rejected from the non‐aggressive ones. Lastly, we expected that perceived victimization would discriminate the rejected‐only students from the rejected‐victimized ones (Schuster, 1999, 2001).

Thus, we further explored whether the self‐reported correlates were consistent with: (1) the profiles derived from the peer‐reported nominations; and (2) the characteristics of the profiles identified in the literature.

## METHODS

### Data and procedure

Data came from the first wave of the Children of Immigrants Longitudinal Survey in Four European Countries (CILS4EU; Kalter, Heath, Hewstone *et al*., 2016). The project was designed to study the intergenerational integration of children of immigrants in a longitudinal research project carried out in England, Germany, Sweden, and the Netherlands. The project surveyed 14–15 year‐old students with and without a migration background in randomly selected classrooms nested in randomly selected schools to draw a sample that was representative of all the school types and regions in each country (Kalter et al., 2016). The students' data were collected via paper and pencil questionnaires administered in the regular national school setting with the support of trained research assistants. The research procedure was consistent with APA ethical standards. Participants and their parents expressed consent to participate in the study and were able to decline participation. Data and additional information are available for download upon request at https://www.cils4.eu/.

### Sample

From the original sample of 18,716 students, we selected a subset of participants according to three criteria: (1) the nominations had to be possible only within the classrooms, restricting the peer status computation at the class level; (2) students in classes whose size were below the first (*n* < =17) decile were dropped. We did this to improve the reliability of the data, given that we considered suspicious the existence of classes made of one person; and (3) Participants' reported gender and year of birth in the first three waves of the CILS4EU project must be consistent over time, to enhance the reliability of the data.

The final sample consisted of 16,224 students, recruited in 800 classes from 432 schools in England (20.7%), Germany (28.3%), Sweden (28.2%), and the Netherlands (22.8%). Approximately half of the participants were male (49.5%), and they were 15.01 years old on average (*SD* = 0.68). Considering the participants' migration background, 47.4% declared their family (i.e., parents and grandparents) was made by only native individuals, 40.5% were born in the survey country but had at least one member of their family who migrated in the survey country, and 10.8% were first‐generation immigrant students (1.3% unknown).

## MEASURES

### Peer assessment indices

Four peer‐reported indices were used to compute the peer status profiles of the students. Originally, the indices indicated the raw number of nominations received by each student. Then, each index was centered on the classroom mean to control for the between‐classroom differences in the means.

Popularity. The *index of popularity* reflected participants' received nominations by other classmates as a popular students in the class (“Who are the most popular students in this class? Here you should write no more than five ID numbers”). The centered index ranged from −4.44 to 22.50 (*M* = 0.00, *SD* = 3.00). The raw index ranged from 0 to 26 (*M* = 1.69, *SD* = 3.15).

Dislike. The *index of dislike* measured the extent to which the participant's classmates would not sit next to him/her. Participants responded to an item asking to write down up to five classmates they did not want to sit by (“Who would you NOT want to sit by? Here you may write down no more than five ID numbers”). The centered *index of dislike* ranged from −4.31 to 20.25 (*M* = 0.00, *SD* = 2.65). The raw index ranged from 0 to 24 (*M* = 1.92, *SD* = 2.81).

Aggression. The *index of aggression* was computed according to the nominations received by participants' classmates as mean to them (“Who is sometimes mean to you?”). Students were not limited in the number of classmates they could list. The centered index ranged from −1.86 to 12.14 (*M* = 0.00, *SD* = 0.83). The raw index ranged from 0 to 13 (*M* = 0.31, *SD* = 0.88).

Victimization. The *index of victimization* measured the nominations received as a victim of the classmates' mean behaviors (“Who are you sometimes mean with?”). Students were not limited in the number of classmates they could list. The centered index ranged from −3.25 to 15.90 (*M* = 0.00, *SD* = 0.93). The raw index ranged from 0 to 17 (*M* = 0.36, *SD* = 1.03).

### Self‐reported correlates of the students' profile

From the wide pool of self‐reported variables measured in the CILS4EU survey (see the online Appendix S1 for the complete questionnaire), we selected a series of social, behavioral, psychological, and well‐being correlates relevant to assessing the construct validity of the profiles.

Intergroup social connections. Participants rated how often they spent time with people from different ethnic groups in the school. Response scales ranged from 1 = “Never/Don't know students from this background” to 5 = “Everyday”. We averaged the items in an *index of intergroup social connection* (*M* = 3.56, *SD* = 1.06) with higher scores indicating a higher number of social connections.

Dating experiences. The number of dating experiences was assessed with the question, “How many boyfriends/girlfriends have you had in the past?” Participants wrote the number of their previous dating experiences. The *index of dating experiences* (*M* = 3.17, *SD* = 4.03) ranged from 0 (never had a boyfriend/girlfriend) to 20 (20 or more boyfriends/girlfriends).

Behavioral problems in school. The scale measured the frequency of participants' behavioral problems in school on a five‐point (range: 1–5) scale (i.e., arguing with the teacher, getting punishment in school, skipping a lesson without permission, coming late to school). We averaged the four items in an *index of behavioral problems in school* (*M* = 1.76, *SD* = 0.67). The index showed an acceptable internal consistency (Cronbach's alpha = 0.70; ω_t_ = 0.71), with higher scores indicating a higher frequency of behavioral problems in school.

Masculinity norms. Three items assessed participants' legitimization of the use of violence (“How much do you agree or disagree with each of these statements? (1) A man should be ready to use violence to defend his wife and children; (2) A man should be ready to use violence against insults; (3) A man should be ready to use violence if someone talks badly about his family”). The response scale ranged from 1 (Strongly disagree) to 5 (Strongly agree) and the averaged *index of masculinity norms* (*M* = 2.77, *SD* = 0.97) showed good reliability (Cronbach's alpha = 0.77; ω_t_ = 0.78).

Perceived victimization. Three items assessed the occurrences of victimization in the last month (“How often have the following things happened in the last month? (1) I was scared of other students; (2) I was teased by other students; (3) I was bullied by other students”). Participants responded on a four‐point Likert scale (1 = Everyday, 2 = Once or several times a week, 3 = Less often, 4 = Never). The three items were averaged in an *index of perceived victimization*, and the responses were re‐coded so that higher scores on the index indicated higher victimization. The index (*M* = 1.29, *SD* = 0.49) showed good internal consistency (Cronbach's *alpha* = 0.77; ω_t_ = 0.78).

Self‐esteem. Four items measured the level of participants' self‐esteem (“How much do you agree or disagree with each of these statements? (1) I have a lot of good qualities; (2) I have a lot to be proud of; (3) I like myself just the way I am; (4) I think things will go well for me in the future). Participants answered on a five‐point Likert response scale (1 = Strongly disagree; 5 = Strongly agree). The averaged *index of self*‐*esteem* (*M* = 4.02, *SD* = 0.65) showed good reliability (Cronbach's *alpha* = 0.81; ω_t_ = .82).

Satisfaction with life. Satisfaction with life (*M* = 7.78, *SD* = 1.94) was measured with a ten‐point Likert single‐items (“On a scale from 1 to 10 where 1 is very unsatisfied, and 10 is very satisfied, how satisfied are you with your life in general?”).

Perceived general health. A single item measured participants' perceived general health compared to others: “How good is your health compared to others of your age?” The response scale ranged from 1 to 5, with a higher score indicating higher general health (*M* = 3.95, *SD* = 0.90).

Socio‐demographic variables. Finally, we considered the gender, age, migration background, and the parents' occupational status. Initially, the occupational status of the mothers and fathers was coded following the *International Socio*‐*Economic Index of Occupational Status* (ISEI; Ganzeboom, 2010). Then, the two ISEI were summed in a *Parents' ISEI* (*M* = 90.12, *SD* = 34.85, range: 23.30–177.66), with a higher score indicating a higher socio‐economic index of participants' parents. We entered these indices and the survey country as control variables in the analyses assessing the construct validity of the profiles.

## STATISTICAL ANALYSES

The latent profile analysis was conducted on the four peer‐reported indices (i.e., popularity, dislike, bullying, and victimization). The best solution for the LPA was determined considering both statistical and theoretical criteria.^1^ The statistical indices represented the starting point for the model selection, but the decision about the appropriate number of classes highly relied on the consistency of the solution with theoretical assumptions, the meaningfulness of the profiles (i.e., interpretability and plausibility of the identified profiles; Geiser, 2010), and the presence of an adequate percentage of participants within each class (Tyndall, Waldeck, Pancani, Whelan, Roche & Pereira, 2018).

After identifying the best LPA solution, we compared the profiles on the self‐reported correlates to identify their characteristics. Given the hierarchical structure of the data with participants nested in the classroom, we considered the two‐level linear mixed models (LMMs), with classrooms as the clustering variable, to be the most appropriate models allowing us to control for the between‐classroom variances of the considered outcomes. The sociodemographic variables (i.e., age, gender, migration background, and parents' ISEI) were inputted as control variables in the LMMs. The LPA was conducted using the statistical software Mplus, version 7 (Muthén & Muthén, 2012), the LMMs were performed using the package *lme4* (Bates, Mächler, Bolker & Walker, 2015) with Satterthwaite's approximation of degrees of freedom (package *lmerTest*; Kuznetsova, Brockhoff & Christensen, 2017) on R‐Studio (vers. 4.2.0). Effect sizes of the main predictor in LMMs were computed following Selya, Rose, Dierker, Hedeker and Mermelstein's (2012) formula based on Nagakawa's conditional *R* ^*2*^ (Nakagawa & Schielzeth, 2013). The Appendix, the supplementary material file, and the analytic code are available at this link www.osf.io/vzwxm/?view_only=d8423e2d69aa469db1a3283e8532beb7.

## RESULTS

### Identification of the peer status profiles

We estimated the LPA models with 2–6 classes. The statistical indices for all the models (named K2 – K6) are reported in Table 2. According to the indices of model fit, each LPA solution with *k* classes had a better fit than the one with *k* − 1 classes. Only relying on the model fit statistics, the solution with six classes was the best one. However, we noticed some relevant issues in the K6 solution. C1_K6_ comprised only 130 (0.8%) participants out of the 16,224 composing the total sample, and we judged this classification implausible due to the very rare incidence of this class in our study population. Moreover, the profiles that emerged from K6 did not add any relevant information compared to K5 (see the online Supplementary Appendix S1). In keeping with this, no more than six classes were estimated because larger solutions would have always yielded at least one class as narrow as C2_K6_. Thus, we decided to reject K6 and evaluate the goodness of K5. The statistical indices of model fit and the quality of the classification supported the selection of the 5‐class solution. The ability to discriminate among different profiles improved compared to K4, with a substantial reduction of the biggest class (from C1_K4_ = 87.2% to C1_K5_ = 83%) and a meaningful participants' distribution in the other classes. Model K5 showed a good classification accuracy, according to the entropy value greater than 0.70 (Meeus *et al*., 2010; Reinecke, 2006), and the classes' posterior probabilities greater than 0.90 (Childs, 2014).

**Table 2:** Results of the latent profile analyses (LPAs)

	nfp	Adj BIC	‐2LL	BLRT *p*	*E*	*n* (%)	PP
K2 (2 classes)	13	232,559	10408.5	<0.001	0.983		
C1_K2_						15,529 (95.7)	0.997
C2_K2_						695 (4.3)	0.947
K3 (3 classes)	18	224,734	7857.1	<0.001	0.988		
C1_K3_						15,350 (94.6)	0.997
C2_K3_						289 (1.8)	0.967
C3_K3_						585 (3.6)	0.951
K4 (4 classes)	23	218,085	6680.8	<0.001	0.972		
C1_K4_						14,142 (87.2)	0.990
C2_K4_						1,292 (8.0)	0.934
C3_K4_						4,893 (3.0)	0.968
C4_K4_						297 (1.8)	0.962
**K5 (5 classes)**	**28**	**213,569**	**4222.1**	**<0.001**	**0.962**		
**C1**_**K5**_						**13,465 (83.0)**	**0.984**
**C2**_**K5**_						**493 (3.0)**	**0.963**
**C3**_**K5**_						**1,282 (7.9)**	**0.934**
**C4**_**K5**_						**235 (1.5)**	**0.965**
**C5**_**K5**_						**749 (4.6)**	**0.910**
K6 (6 classes)	33	210,087	3514.7	<0.001	0.965		
C1_K6_						130 (0.8)	0.984
C2_K6_						13,194 (81.3)	0.983
C3_K6_						1,198 (7.4)	0.933
C4_K6_						768 (4.7)	0.949
C5_K6_						717 (4.4)	0.912
C6 _K6_						217 (1.4)	0.966

*Notes*: The selected solution is reported in bold. Nfp = numbers of free parameters; Adj BIC = sample‐size adjusted Bayesian Information Criterion; −2LL = 2‐times log‐likelihood difference; BLRT *p* = *p*‐value of the bootstrapped likelihood ratio test; *E* = entropy; n (%) = number and percentage of participants in the class; PP = posterior probability.

**Fig. 1:**
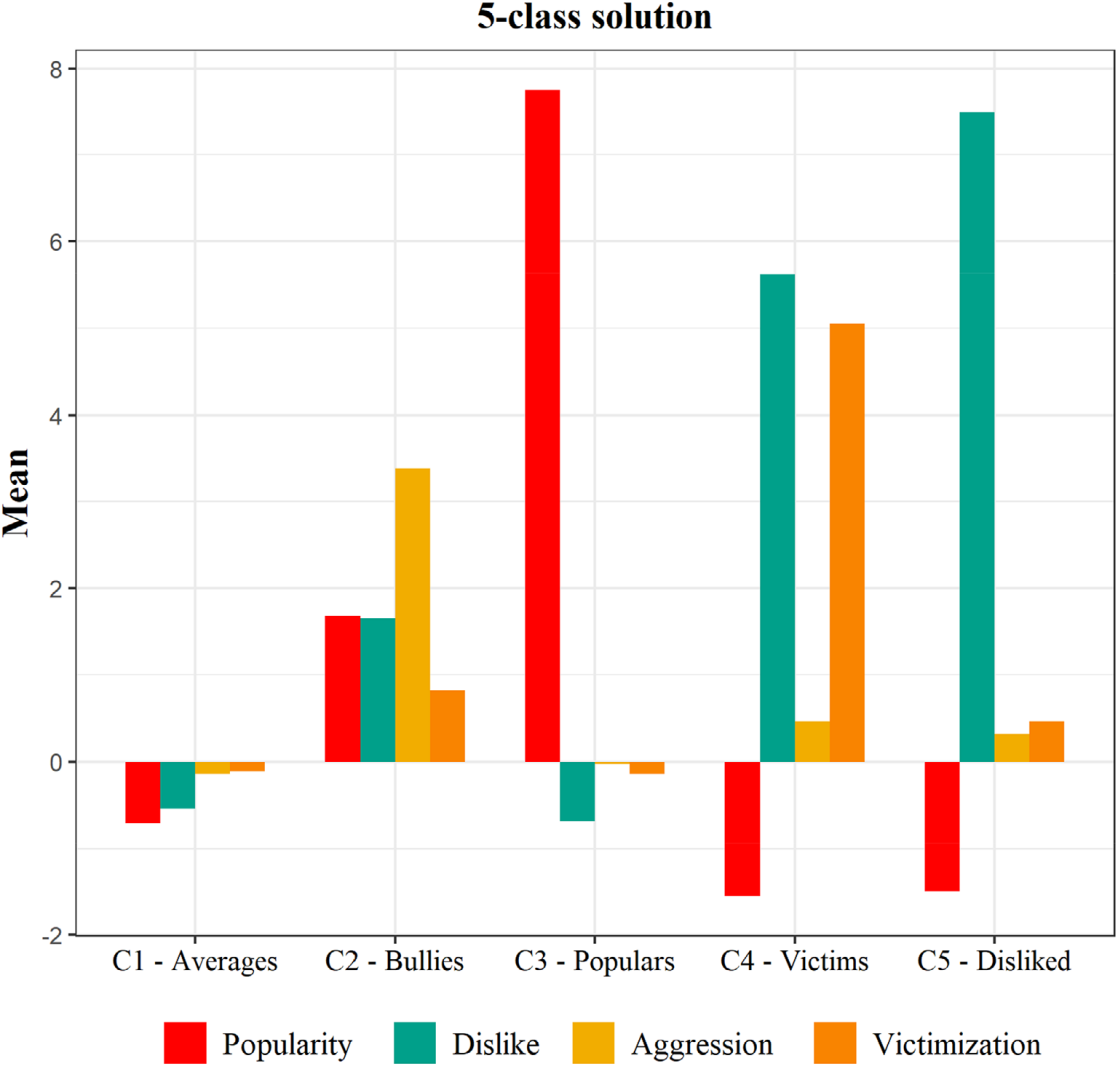
Graphic representation of the 5‐class solution (Model K5): classroom‐centered, mean values of the peer‐reported sociometric indices are displayed.

The estimated means of the peer status indices and dimensions for each of the five classes of the model K5 are depicted in Fig. 1 and presented in Table 3. The five profiles appeared reasonable and theoretically plausible.

**Table 3:** Classroom‐centered estimated means (SD) and gender distribution of the sociometric indices and dimensions for the 5‐class solution (model K5)

K5	Popularity	Dislike	Aggression	Victimization	Gender (males)
C1_K5_ – *Averages*	−0.71 (1.63)	−0.54 (1.64)	−0.14 (0.45)	−0.11 (0.57)	48.0%
C2_K5_ – *Bullies*	1.68 (3.85)	1.65 (3.06)	3.38 (1.50)	0.82 (1.27)	74.4%
C3_K5_ – *Populars*	7.75 (2.84)	−0.69 (1.89)	−0.02 (0.66)	−0.14 (0.61)	55.4%
C4_K5_ – *Victims*	−1.55 (1.52)	5.62 (4.46)	0.46 (1.10)	5.05 (2.36)	66.4%
C5_K5_ – *Disliked*	−1.49 (1.25)	7.49 (2.81)	0.31 (0.60)	0.46 (0.85)	54.3%

Participants in the largest class C1_K5_ scored slightly below the classroom mean on all the peer status indices. They presented no high or low peaks in any of the sociometric indices; thus, given that they were the great majority, we considered this group of students as the *averages*.

Students in the C2_K5_ showed the highest mean value of the *index of aggression*, both compared to the other peer status indices within this class and compared to the score of this index in the other classes. Thus, this class represented the profile of the *bully*. We also observed that the bullies' scores on the *index of popularity, dislike*, and (slightly) *victimization* were above the average classroom score, indicating that peers considered bullies as popular, but they also disliked and victimized them more than average.

We coded the students in class C3_K5_ as *populars*. They showed the highest score on the *index of popularity* across the other classes and indices within the class C3_K5_. They also presented approximately the same values on the *index of victimization* and *aggression* of the other classmates and scores on the *index of dislike* slightly below the classroom mean.

C4_K5_ represented the *victims*, as their score on the *index of victimization* was the highest across the other classes of the model K5. These students were also highly excluded by other classmates, they were slightly more aggressive to other classmates compared to the other students in the class, and their classmates considered them less popular than the others.

Lastly, C5_K5_ represented the disliked students. They showed the highest level of the *index of dislike* both within the class itself and among the other classes. Their scores on the *index of aggression* and *victimization* aligned with the average scores of the other classmates, and they were considered less popular than their classmates.

The analysis of gender distribution showed that males and females were rather equally spread among the averages, popular, and disliked. Differently, the bullies and the victims (the profiles characterized by high acted or received aggression) were largely composed of males.

### Self‐reported correlates of the peer status profiles

**Fig. 2:**
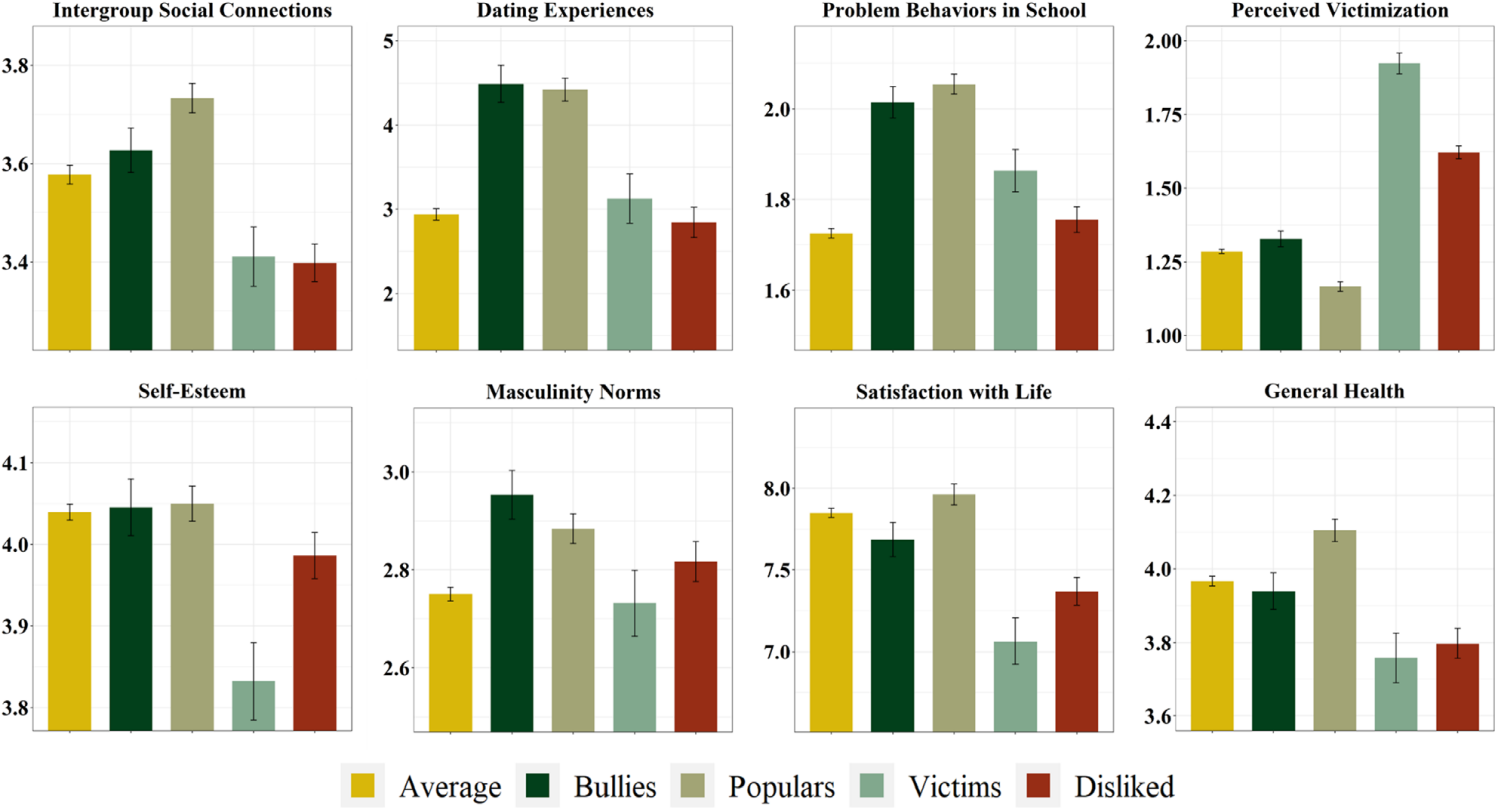
Profiles' estimated means and standard errors of the self‐reported correlates.

All the statistical parameters of the models tested are reported in Table 4 and Fig. 2 displays the profiles' estimated means and standard errors on the self‐reported indices. The results are discussed below. As general considerations, we observed that the profile categorization was a significant predictor of all the self‐reported correlates, meaning that the profiles significantly differed from each other on the self‐reported correlates (even if with a small effect size 0.02 < Cohen's *f* < 0.10). Also, the indices' variance explained at the classroom level was significant for all the models, confirming the adequacy of the random‐intercept modeling. We also observed that the effects of the sociodemographic variables were significant in almost all the models, underlining the need to control for these variables.

**Table 4:** Results of the LMMs testing the differences among students' profiles on the self‐reported correlates

		F (DFnum, DFden)						
Indices	Class – level variance	K5	Gender	Age	Migration background	Parents' ISEI	Country	Cohen's *f* (K5)
Intergroup social connections	0.19***	19.82*** (4, 10,599)	22.06*** (1, 10,918)	6.33* (1, 10,936)	30.62*** (2, 10,985)	7.26** (1, 11,086)	421.29*** (3, 784)	0.08
Dating experiences	0.99***	43.70*** (4, 10,000)	254.13*** (1, 10,183)	39.68*** (1, 9,368)	1.11 (2, 10,122)	17.29*** (1, 9,455)	8.02*** (3, 718)	0.07
Behavioral problems in school	0.03***	77.86*** (4, 10,939)	92.17*** (1, 11,099)	38.58*** (1, 11,089)	14.10*** (2, 11,029)	2.24 (1, 10,410)	108.87*** (3, 794)	0.09
Perceived victimization	0.01***	159.21*** (4, 11,029)	2.04 (1, 10,528)	10.19** (1, 10,541)	11.93*** (2, 10,386)	0.70 (1, 8,594)	94.64*** (3, 749)	0.09
Self‐esteem	0.02***	5.79*** (4, 10,966)	488.30*** (1, 10,788)	0.08 (1, 10,774)	36.54*** (2, 10,656)	12.74*** (1, 9,315)	94.81*** (3, 716)	0.02
Masculinity norms	0.03***	9.08*** (4, 10,857)	1320.03*** (1, 10,613)	2.06 (1, 10,608)	16.87*** (2, 10,496)	110.01*** (1, 9,083)	27.80*** (3, 754)	0.04
Satisfaction with life in general	0.07***	19.24*** (4, 11,015)	276.54*** (1, 10,481)	19.94*** (1, 10,480)	0.57 (2, 10,349)	0.97 (1, 8,607)	38.60*** (3, 791)	0.03
General health	0.01***	12.83*** (4, 11,022)	104.12*** (1, 10,373)	3.18 (1, 10,390)	4.32* (2, 10,243)	16.62*** (1, 8,298)	24.63*** (3, 801)	0.02

	Mean (SE)							
	*Averages*	*Bullies*		*Populars*	*Victims*		*Disliked*	
Intergroup social connections	3.58_a_ (0.02)	3.63_a,b_ (0.05)		3.73_b_ (0.03)	3.41_c_ (0.06)		3.40_c_ (0.04)	
Dating experiences	2.94_a_ (0.07)	4.49_b_ (0.22)		4.42_b_ (0.14)	3.13_a_ (0.29)		2.84_a_ (0.19)	
Behavioral problems in school	1.72_a_ (0.01)	2.01_c,d_ (0.04)		2.05_c_ (0.02)	1.86_b,d_ (0.05)		1.76_a,b_ (0.03)	
Perceived victimization	1.28_a_ (0.01)	1.33_a_ (0.03)		1.17_b_ (0.02)	1.92_c_ (0.04)		1.62_d_ (0.02)	
Self‐esteem	4.04_a_ (0.01)	4.05_a_ (0.04)		4.05_a_ (0.02)	3.83_b_ (0.05)		3.99_a_ (0.03)	
Masculinity norms	2.75_a_ (0.01)	2.95_b_ (0.05)		2.88_b_ (0.03)	2.73_a,b_ (0.07)		2.82_a,b_ (0.04)	
Satisfaction with life in general	7.85_a_ (0.03)	7.69_a,b_ (0.10)		7.96_a_ (0.06)	7.06_b,c_ (0.14)		7.37_b_ (0.08)	
General health	3.97_a_ (0.01)	3.94_a,c_ (0.05)		4.10_b_ (0.03)	3.76_c_ (0.07)		3.80_c_ (0.04)	

*Notes*: Values with different subscripts are significantly (*p* < 0.05; Bonferroni adjustment) different from each other.

* *p* < 0.05,

** *p* < 0.01,

*** *p* < 0.001.

## DISCUSSION

In this study, we aimed at identifying a peer status classification integrating previous findings on the students' peer status. Indeed, the current knowledge of the students' peer status relies on different research traditions that identified a scattered constellation of profiles partially overlapping. To clarify this confusing scenario, we saw the urge to identify a classification capturing the communal explanation of the different considered dimensions and mapping in a unified classification all the peer status profiles. We used the four dimensions of popularity, aggression, dislike, and victimization to classify via a latent profile analysis (LPA) the peer status of 16,224 young European adolescents. Also, we observed the social, psychological, cognitive, behavioral, and well‐being correlates of the profiles to assess their construct validity. The analysis identified five profiles: the popular, bully, disliked, victim, and average students. Overall, this work adds to the peer‐status research as it sought to test and replicate on a large sample of early adolescent students from four European countries the existence of the peer‐status profiles identified comprehensively across different peer‐status research traditions, by using an advanced analytical strategy also considering diverse self‐reported correlates to validate the profiles.

The *popular*‐*nonaggressive* students matched with the nonaggressive subtypes of the populars (i.e., prosocial and model) and the prosocial dominant students from the evolutionary tradition. They were considered highly popular by their classmates, they had low chances of being disliked, and were not aggressive toward others, nor they were victims of others' aggression. According to the self‐reported correlates, these students had more frequent dating experiences and *intergroup* social connections than the other profiles, in line with both the affiliative characteristics consistently depicted in the prosocial subtypes of the populars. They also reported the highest general health, aligning with previous findings showing that respect and admiration in face‐to‐face groups predicted increased subjective well‐being (Anderson *et al*., 2012). Together with the bullies, the populars also showed the highest behavioral problems in school (Mayeux *et al*., 2008) – typically observed in the broader profile of the perceived popular students (Adler & Adler, 1998). Based on the theoretical introduction and the considered self‐reported correlates, the results hint that the profile of the popular students would also overlap with the well‐liked students from the sociometric tradition. However, with the present findings, we could not claim empirical support for this proposition, given that the dataset we used did not measure likability.

The *bully* students showed the peer‐reported characteristics typically observed in the controversial, aggresive subtypes of populars (populistic and tough), and coercive‐dominants, as well as in the aggressive rejected students. Indeed, they were distinguished from their classmates because of their highest levels of aggression. However, they also received a high number of nominations on the indices of both popularity and dislike (similar to both the highly liked and disliked sociometric controversial students). Therefore, the profile of the bully included the characteristics of both the aggressive popular and the aggressive rejected students. Indeed, they possessed the popular high status observed in the aggressive subtypes of developmental populars and the evolutionary bully (Hawley, 1999; Rodkin *et al*., 2000) Also, they were also occasionally victims of others' aggression – consistently with the findings that bullies can likely be also excluded and victimized by others (i.e., bully‐victim; Perren & Alsaker, 2006; Solberg *et al*., 2007; Veenstra *et al*., 2005; Farmer *et al*., 2010).

The self‐reported correlates supported the face validity of the bully profile, given that bullies showed higher aggressive masculinity norms than the average students (even if not *significantly* higher than the populars, victims, and the disliked). As for the popular students, they showed high levels of intergroup social connections (even if not significantly higher than the averages) and the highest number of dating experiences, indicating the high status and influence of both the two profiles students (Ibarra & Andrews, 1993; Sprecher & Regan, 2002).

The two low‐status profiles matched Schuster's (1999) distinction between the rejected‐only and the rejected‐victimized profiles: the *disliked* only received a high amount of dislike nominations, whereas the *victims* were both disliked and victims of the peers' bullying. Also, these two profiles could match with the withdrawn subtypes of rejected students (Inderbitzen *et al*., 1997), given that they showed a score of aggression similar to the average students. Consistently with previous studies emphasizing the low‐accepted students' psychological maladjustment (Ollendick *et al*., 1992; Parker & Asher, 1987), the *disliked* and *victims* showed the lowest perceived general health and satisfaction with life. Their self‐reported correlates showed that they had the lowest number of *intergroup* social connections and that the victims perceived more victimization than the disliked and all the other profiles. The victims also had the lowest self‐esteem, aligning with research observing low self‐esteem in victims of bullies (O'Moore & Kirkham, 2001). These results pointed to the victims as the peer status prototype presenting the worst implications in terms of psychological health (Rigby, 2001).

Finally, the present classification did not identify the sociometric category of the neglected students. It can be that, given the presumed overlap between the neglected and average students (Hill & Merrell, 2004; Gifford‐Smith & Brownell, 2003; Brown, 2015), the LPA classification – more accurate than the traditional use of cutoffs – would reveal the meaninglessness of the neglected category categorizing them into the profile of the *average* students, whose peer‐ and self‐reported attributes were around the average classroom score on all the considered indices.

Overall, the multidimensional classification led to the identification of a set of profiles that summarized the scattered constellation of the profiles found in different research traditions. Besides the sophisticated statistical technique used to derive the peer status profiles (i.e., LPA), these results are further strengthened by the large and international sample they drew on, and by the consistency found between the peer‐ and self‐reported characteristics of the profiles.

The conceptual validity of the present findings is supported by their theoretical alignment with the recent proposition of *prestige* and *dominance* as fundamental dimensions of human social status (Cheng, Tracy, Foulsham, Kingstone & Henrich, 2013; Henrich & Gil‐White, 2001). Prestige and dominance are considered as two independent strategies, ubiquitous in the social groups, that promote patterns of behaviors useful in attaining high social rank, reflecting the power of influence one possesses within the group (Berger, Rosenholtz & Zelditch, 1980; Cheng & Tracy, 2014). Dominant individuals attain high status through the induction of fear, threats, coercion, and imposition. Differently, prestigious individuals' high status is genuinely conferred from the subordinates, who seek access to those recognized as the most skilled and worthy‐of‐emulation members of the group, in order to learn from them and to benefit from their resources (Cheng & Tracy, 2014; Maner & Case, 2016). Prestigious individuals were described as well‐liked, cooperative, and possessing high social skills. The dominant were highly aggressive, arrogant, and low agreeable, behaving antisocially (Van Kleef, Homan, Finkenauer, Gündemir & Stamkou, 2011), with both the profiles showing athletic abilities, leadership, and social influence (Cheng, Tracy & Henrich, 2010). The fact that the characteristics of the popular and bully students corresponded to the description of the prestigious and dominant individuals in the evolutionary literature (Cheng *et al*., 2010), strengthened the conceptual reliability of the presented classification. On the one hand, the convergence of the findings supports the conceptual validity of the identified profiles; on the other, it suggests the application of the prestige‐dominance account in the context of peer status in future research.

This work could contribute to peer status research by highlighting that an LPA‐based classification considering multiple dimensions of peer status from the different research traditions could heuristically integrate the scattered literature of the peer status prototypes into a unified classification. Indeed, the present work's simultaneous focus on the four dimensions of popularity, aggression, disliked, and victimization overcame the limitation of the fragmented research traditions that considered these dimensions separately. However, future works should also include further important traditionally used dimensions (e.g., likeability, social exclusion) that could yield an even more comprehensive classification. Also, the present research confirmed the already reported limitations of the traditional cut‐off‐based classification (Hubbard *et al*., 2013), suggesting the use of LPA as a new standard in peer status research.

### Limitations and future research

Despite the profile of the popular theoretically matched with the sociometric profile of the well‐liked students, future studies should also consider how a multidimensional LPA classification would emerge also considering likeability. Several studies showed that likeability and popularity are different constructs even if they tend to be moderately (*r =* 0.47; van den Berg, Lansu & Cillessen, 2020) and highly related (Brand & Mesoudi, 2019), with correlations' coefficient around 0.70–0.80 in the early adolescence (Cillessen & Mayeux, 2004; Ilmarinen *et al*., 2019). However, the data we analyzed did not consider the peer‐reported index of likeability. Future research needs to include peer‐reported likability to claim support for the overlap between the popular profile of the present classification with the sociometric well‐liked ones.

Besides the lack of a measure for liking, another limit on the peer‐assessment indices concerns the lack of a behavioral index assessing the actual social exclusion of the disliked students. For this reason, we decided to label the profile that received the highest dislike nomination as disliked, rather than *rejected* as done in the traditional sociometric classification (Coie *et al*., 1982). Indeed, it must be noted that the index of dislike assessed classmates' behavioral intentions to not sit by the participants that we considered a proxy for disliking and that could be only moderately related to actual exclusionary behaviors (Sheeran, 2002). Therefore, although even the traditional sociometric literature (Coie *et al*., 1982) called such highly disliked students *rejected*, we opted to name them disliked to provide a results interpretation the closest possible to the empirical measures. However, future research should consider adding a more direct measure of behavioral exclusion as a dimension of the peer status classification. In doing so, the classification could test whether the disliked students are in fact those also targeted with social exclusion, therefore confirming their alignment with the rejected‐only profiles identified by Schuster (1999, 2001).

Also, the current classification did not identify a separate profile matching exactly with the aggressive rejected students (Inderbitzen *et al*., 1997). Rather, the peer‐reported characteristics of the aggressive rejected were merged into the profile of the bullies, who also displayed moderate levels of popularity. Differently, the withdrawn rejected could be recognized within the victims and the excluded profiles, who were rejected students not displaying aggressive tendencies. However, this interpretation cannot be considered conclusive given that the dataset did not include self‐reported measures of social withdrawal. Therefore, future research should replicate if the aggressive rejected students and the bullies overlap in classification based on the peer‐reported indices of popularity, aggression, and rejection. Research should also include peer‐ or self‐reported indices able to identify the withdrawn and aggressive rejected among the low‐status profiles.

Despite the strength of the LPA, the findings have some analytical shortcomings that must be considered. For instance, the sample was not representative of the countries participants came from. It must be noted that, given that the CILS4EU project's primary focus was on the children of immigrants, the original authors decided to oversample schools with high immigrant proportions. However, we do not expect that this issue would bias the student's classification or the analysis of the correlates. Indeed, despite the oversampling strategy, the overall sample was still well balanced between native, and children of immigrant students, and we considered the migration background as a control variable in the investigation of the profiles' correlates.

Another limitation of the present study is that it did not consider peer‐ or self‐reported indices validly measuring prosocial behaviors. Therefore, the present work could not test the prediction that the populars are cooperative and helpful toward others. Moreover, our classification did not speak about other known roles in the peer dynamics (e.g., reinforcers or defenders of the bullies; Farmer *et al*., 2010). Future studies should address these issues by further examining how the bullying‐related roles could shape the classification of the students' social status, perhaps identifying additional dimensions that can account for these roles. Moreover, besides the validation that the victims perceived higher victimization than the excluded‐only students and that the victims showed lower self‐esteem than the excluded‐only, we could not identify what distinguished the two groups, given that they were very similar on all the other self‐reported correlates. Future research should focus on other variables (e.g., personality traits) to identify key characteristics distinguishing the two low‐status profiles. Although the LPAs separately conducted in the different countries yielded similar results justifying their aggregation in a whole sample (see the Supplementary Material file) and the LMMs controlled for the effect of the country, future research could better investigate possible cultural differences in the peer‐status profiles. Lastly, future research can also try to address some of the apparently conflicting results of this work. We found that the popular students showed some of the antisocial behaviors (i.e., behavioral problems in school) typical of the broader sociological perceived popular students (Adler *et al*., 1992), but not found in the prosocial subtypes of popular students (e.g., Rodkin *et al*., 2000). It could be that in adolescence those with higher popularity – regardless if attained with or without aggressivity – would normatively show some rebellious and antisocial behaviors, but future studies needs to verify this claim.

## CONCLUSION

This work aimed at synthesizing the different profiles identified in the sociometric, sociological, and evolutionary research traditions in a multidimensional classification considering popularity, aggression, dislike, and victimization as core dimensions of qualifying students' interpersonal relationships. The use of latent class analysis that relied on statistical indices rather than on a priori cutoffs, besides the use of a large and international sample, supported the theoretical contributions of this work with methodological reliability. This work sought to reawaken the peer status research resolving past inconsistent findings on the students' peer‐group dynamic and highlighting the importance of simultaneously considering multiple dimensions identified in the different research traditions to base the peer status classification. The multidimensional classification can have applied implications in research and interventions, being it a reliable tool for the identification of those at the utmost risk of being victimized, as well as the key actors to target for the promotion of group cohesion.

## Supporting information


**Appendix S1** Supporting Information.

## Data Availability

Data and additional information are available for download upon request at https://www.cils4.eu/.

## Endnote

^1^
To assess the goodness of fit of the models, we considered that: (1) the sample‐size adjusted Bayesian information criterion (BIC) for solutions with *k* classes should be at least ten points lower than solutions with *k* – 1 classes (Kass & Raftery, 1995); (2) solutions with entropy; and (3) classes' posterior probabilities closer to 1 should be preferred as they indicate a better quality of the classification (Reinecke, 2006). We also considered (4) the bootstrapped likelihood ratio test (BLRT). A significant *p*‐value of the 2‐times log‐likelihood difference (−2LL) tested by the BLRT indicated a statistically significant improvement in the model fit due to the estimation of an additional class (Lo, Mendell & Rubin, 2001).
