# Supplementary material for: Exploring the peer status prototypes: A large‐scale latent profile analysis on high‐school students from four European countries
Source: Scand J Psychol. 2022 Aug 8;64(1):40–52. doi: 10.1111/sjop.12863 (PMC10087329; doi:10.1111/sjop.12863)

**Supplementary Material**

**The 6-class solution**

Although the 6-class solution showed better statistical indices than the 5-class solution, the identified profiles were less meaningful than the 5-class solution. Table 1S and figure 1S describe the characteristics of the profiles based on their scores on the peer-nomination indices of popularity, dislike, aggression, and victimization.

Table 1S. Mean on the nomination indices of the 6-class profiles

| K6 | *Popularity* | *Dislike* | *Aggression* | *Victimization* |
| --- | --- | --- | --- | --- |
| C1_K6_ | 7.865 | -0.727 | -0.097 | -0.147 |
| C2_K6_ | 3.346 | 3.236 | 5.403 | 1.300 |
| C3_K6_ | -0.709 | -0.543 | -0.175 | -0.124 |
| C4_K6_ | 0.872 | 0.756 | 2.121 | 0.547 |
| C5_K6_ | -1.499 | 7.591 | -0.065 | 0.054 |
| C6_K6_ | -1.590 | 5.796 | 0.359 | 5.132 |

Figure 1S. 6-class solution


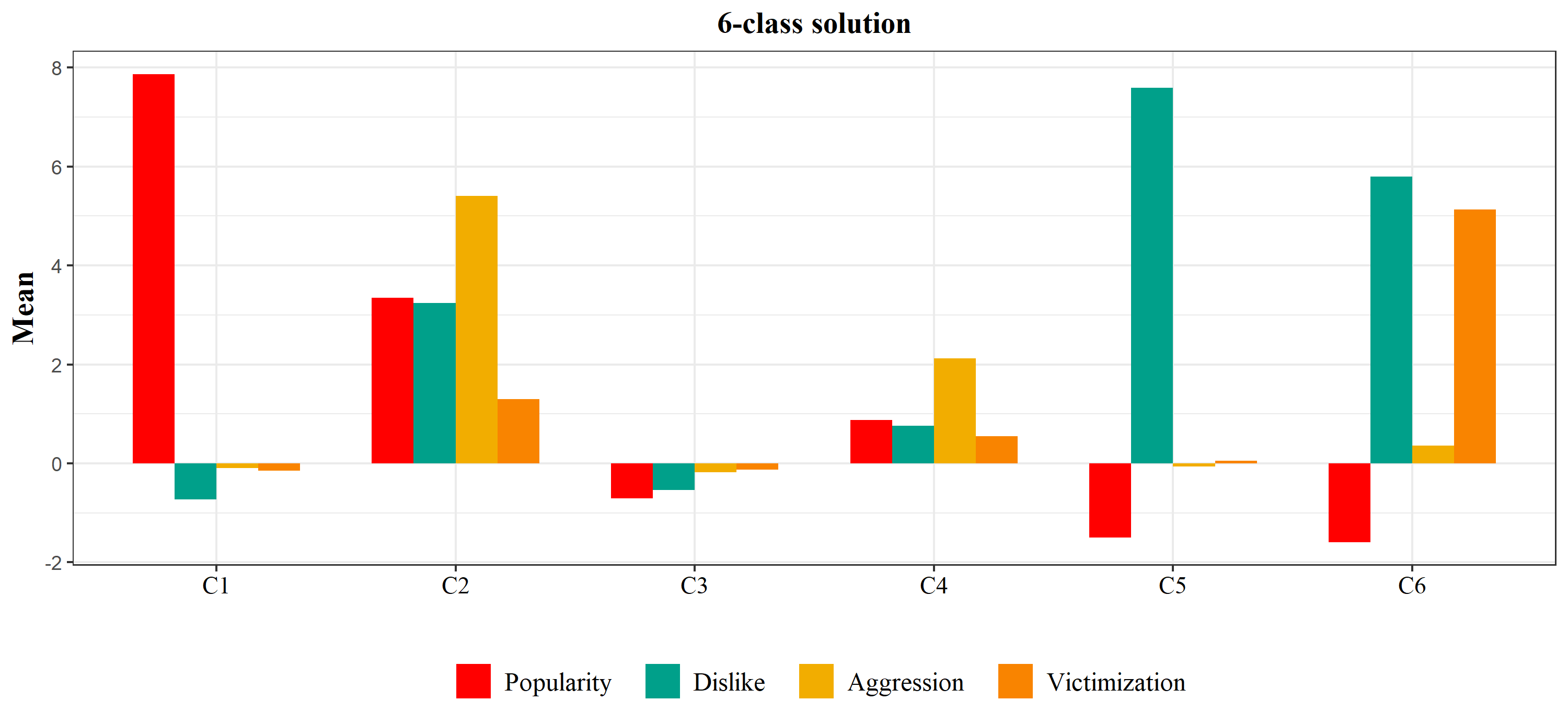


The 6-class solution differed from the 5-class ones because the former further split the profile of the bullies into two subtypes (C2 and C4). These two classes showed the exact pattern of scores with the only difference that C2 presented on average higher mean scores on all the four indices than C4. It meant that the two classes only differed quantitatively on the number of nominations received, not qualitatively on their characteristics. To our consideration, this did not match with any of the previous peer-status profiles identified in the literature, nor was relevant in terms of understanding the peer-status dynamics. Moreover, given that the bully profile from the 5-class solution showed the same pattern of C2 and C4, with its score on the indices being in between C2 and C4, and that the model with 5-classes was statistically more parsimonious (with its students’ distribution over the 5 prototypes being more balanced and meaningful), we opted for the 5-class solution as the best one and did not further LPA with more than six classes.

**The 5-class solution by country**

The LPA was conducted within each country, extracting five classes to investigate overlaps and differences with the 5-class solution on the entire sample. Table 2S reports statistical indices and the number of individuals in each class within each country supporting the selection of 5 classes in all the countries. Indeed, the 5-classes solutions showed good entropy and classes’ posterior probabilities. Figure 2Sa-b displays the profiles. The primary set of profiles of the average, popular, bullies, disliked, and victims students from the whole sample were largely replicated in the analyses on the separated countries. Specifically, the average and popular profiles were both found in the overall sample and each country. Concerning bullies and victims, the country-specific profiles showed the same pattern of peer nomination scores as the whole sample solution, even if we observed slight differences in the absolute levels of each index (*e.g.,* dislike of Dutch bullies is higher than German ones). The only substantial difference is that the disliked students were not identified in the Netherlands and Sweden. As shown in Figure 2b, it was likely due to the disliked profile spread among the bullies and the victims. Indeed, the dislike index was higher for the bullies and victims from Sweden and The Netherlands than those from Germany and England. Also, in The Netherlands, the solution identified two subtypes of bullies who differ only quantitatively on the indices. In Sweden, the solution identified two subtypes of victims.

|  | **nfp** | **Adj BIC** | **-2LL** | **BLRT *p*** | ***E*** | **n (%)** | **PP** |
| --- | --- | --- | --- | --- | --- | --- | --- |
| England | 28 | 43786 | 745.5 | < .001 | .962 |  |  |
| C1_ENG_ |  |  |  |  |  | 2703 (80.4) | .985 |
| C2_ENG_ |  |  |  |  |  | 136 (4.0) | .946 |
| C3_ENG_ |  |  |  |  |  | 297 (8.8) | .948 |
| C4_ENG_ |  |  |  |  |  | 120 (3.5) | .928 |
| C5_ENG_ |  |  |  |  |  | 105 (3.1) | .913 |
| Germany | 28 | 69645 | 1178.8 | < .001 | .938 |  |  |
| C1_GER_ |  |  |  |  |  | 3559 (77.6) | .970 |
| C2_GER_ |  |  |  |  |  | 176 (3.8) | .936 |
| C3_GER_ |  |  |  |  |  | 447 (9.7) | .917 |
| C4_GER_ |  |  |  |  |  | 118 (2.5) | .961 |
| C5_GER_ |  |  |  |  |  | 286 (6.2) | .886 |
| Netherland | 28 | 46186 | 1287.7 | < .001 | .979 |  |  |
| C1_NET_ |  |  |  |  |  | 3156 (85.3) | .992 |
| C2_NET_ |  |  |  |  |  | 38 (1.0) | .984 |
| C3_NET_ |  |  |  |  |  | 322 (8.7) | .940 |
| C4_NET_ |  |  |  |  |  | 44 (1.2) | .995 |
| C5_NET_ |  |  |  |  |  | 140 (3.8) | .984 |
| Sweden | 28 | 40034 | 1761.7 | < .001 | .982 |  |  |
| C1_SWE_ |  |  |  |  |  | 3928 (85.8) | .993 |
| C2_SWE_ |  |  |  |  |  | 99 (2.2) | .964 |
| C3_SWE_ |  |  |  |  |  | 328 (7.2) | .940 |
| C4_SWE_ |  |  |  |  |  | 199 (4.3) | .988 |
| C5_SWE_ |  |  |  |  |  | 23 (0.5) | 1.00 |

Table 2S. Latent Profile Analyses (LPAs) 5-class solution for the four countries.

*Note.* The selected solution is reported in bold. nfp = numbers of free parameters; Adj BIC = sample-size adjusted Bayesian Information Criterion; -2LL = 2-times log-likelihood difference; BLRT *p* = *p-*value of the bootstrapped likelihood ratio test; *E* = entropy; n (%) = number and percentage of participants in the class; PP = posterior probability.

Figure 2a. 5 classes solution of LPAs on English and German samples


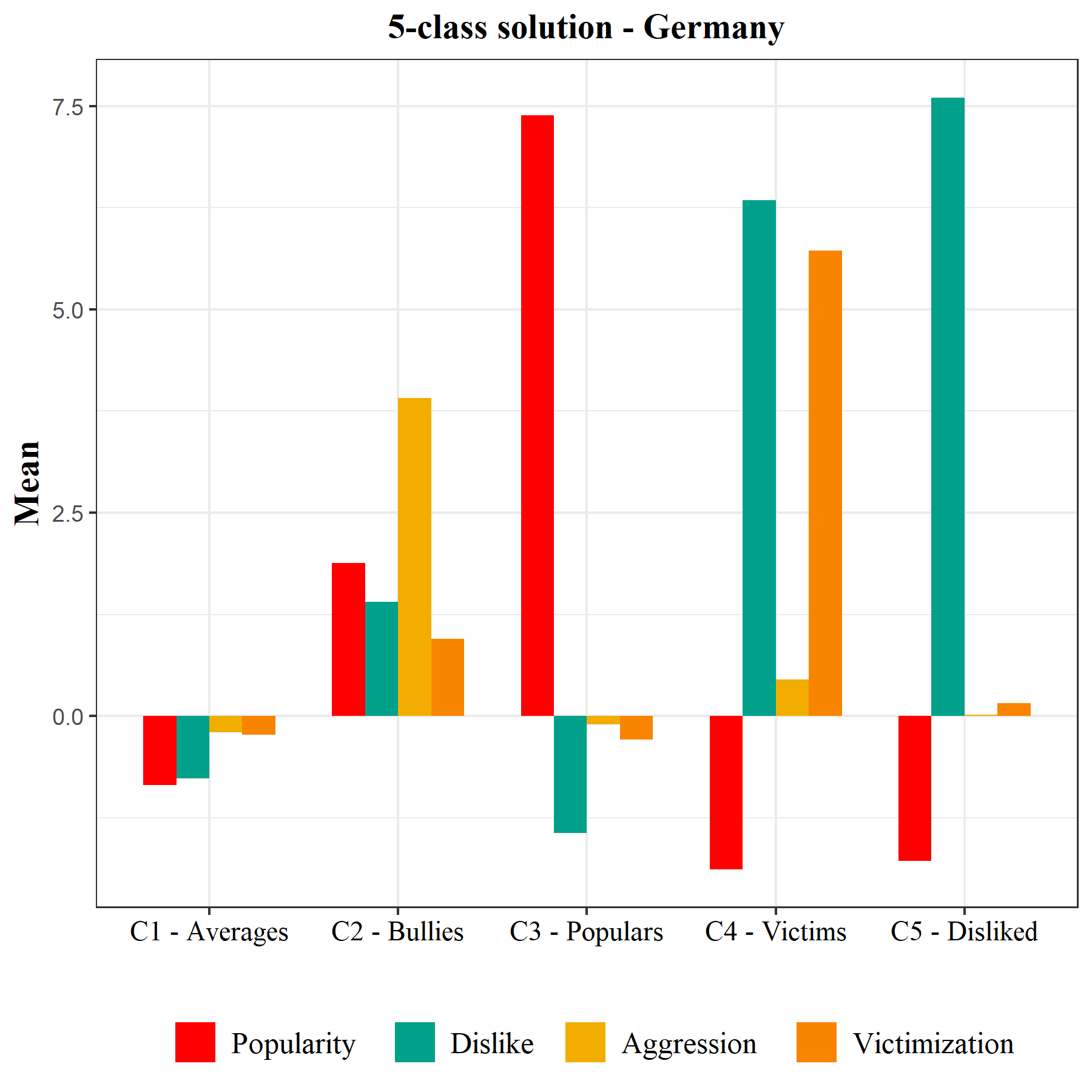

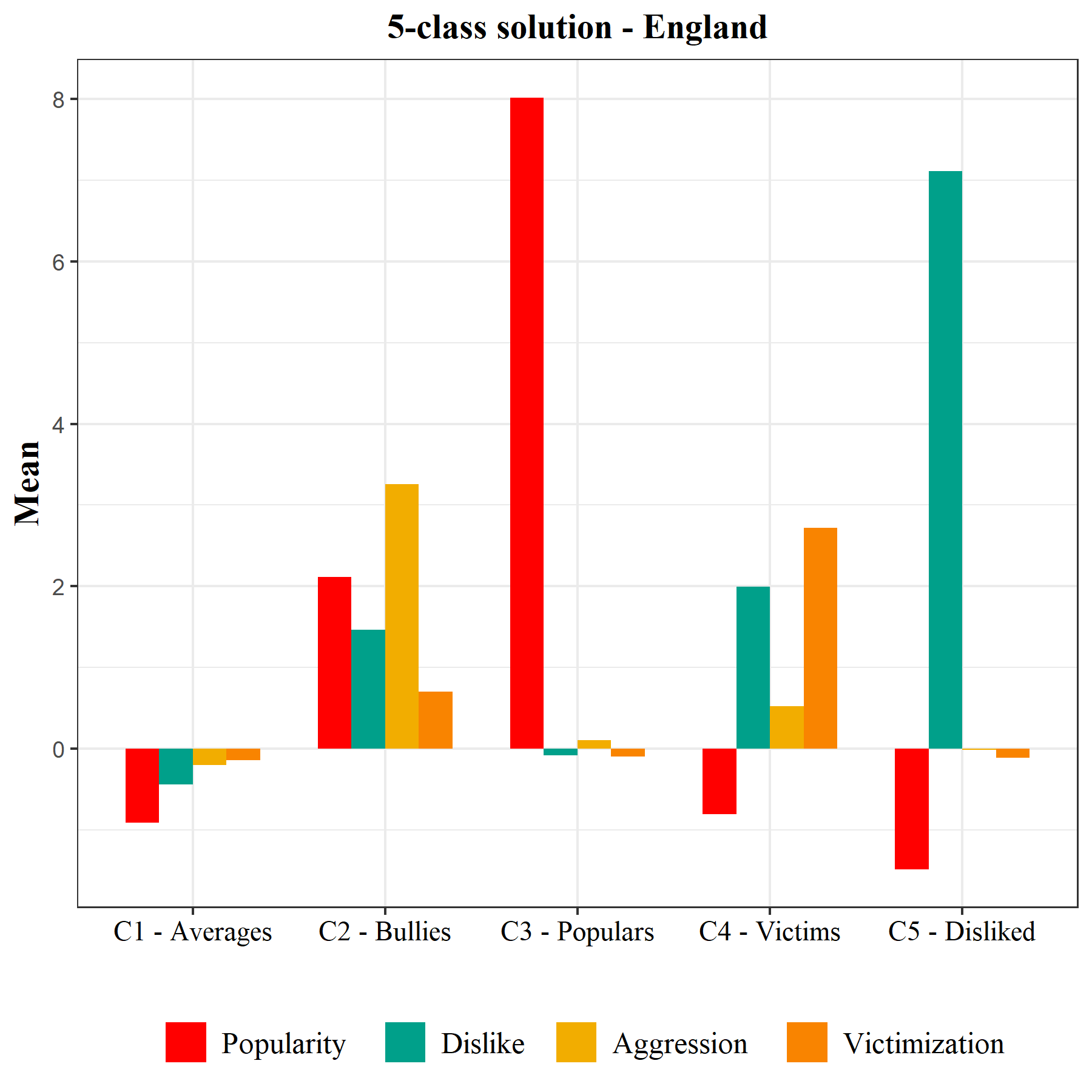


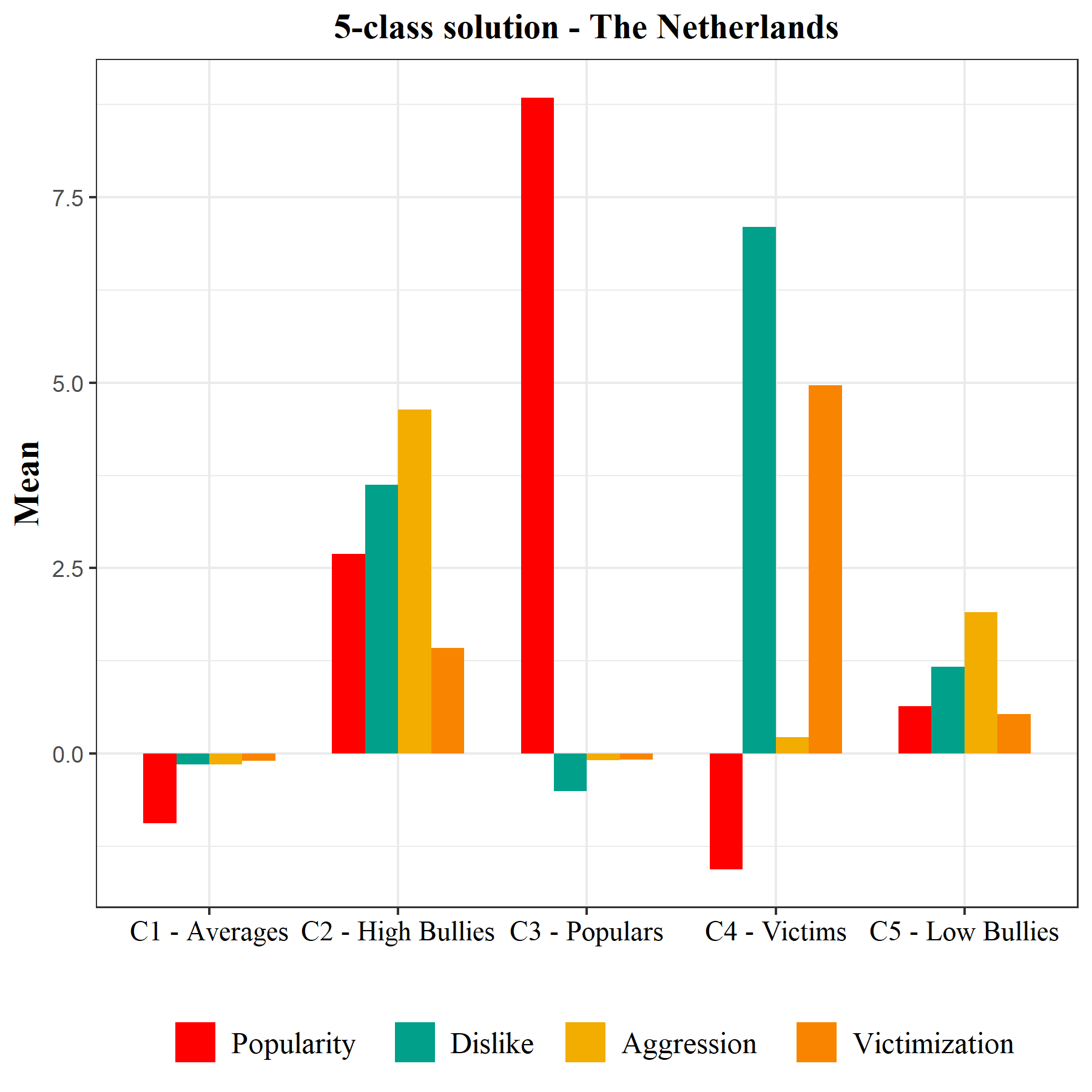
Figure 2b. 5 classes solution of LPAs on Dutch and Swedish samples


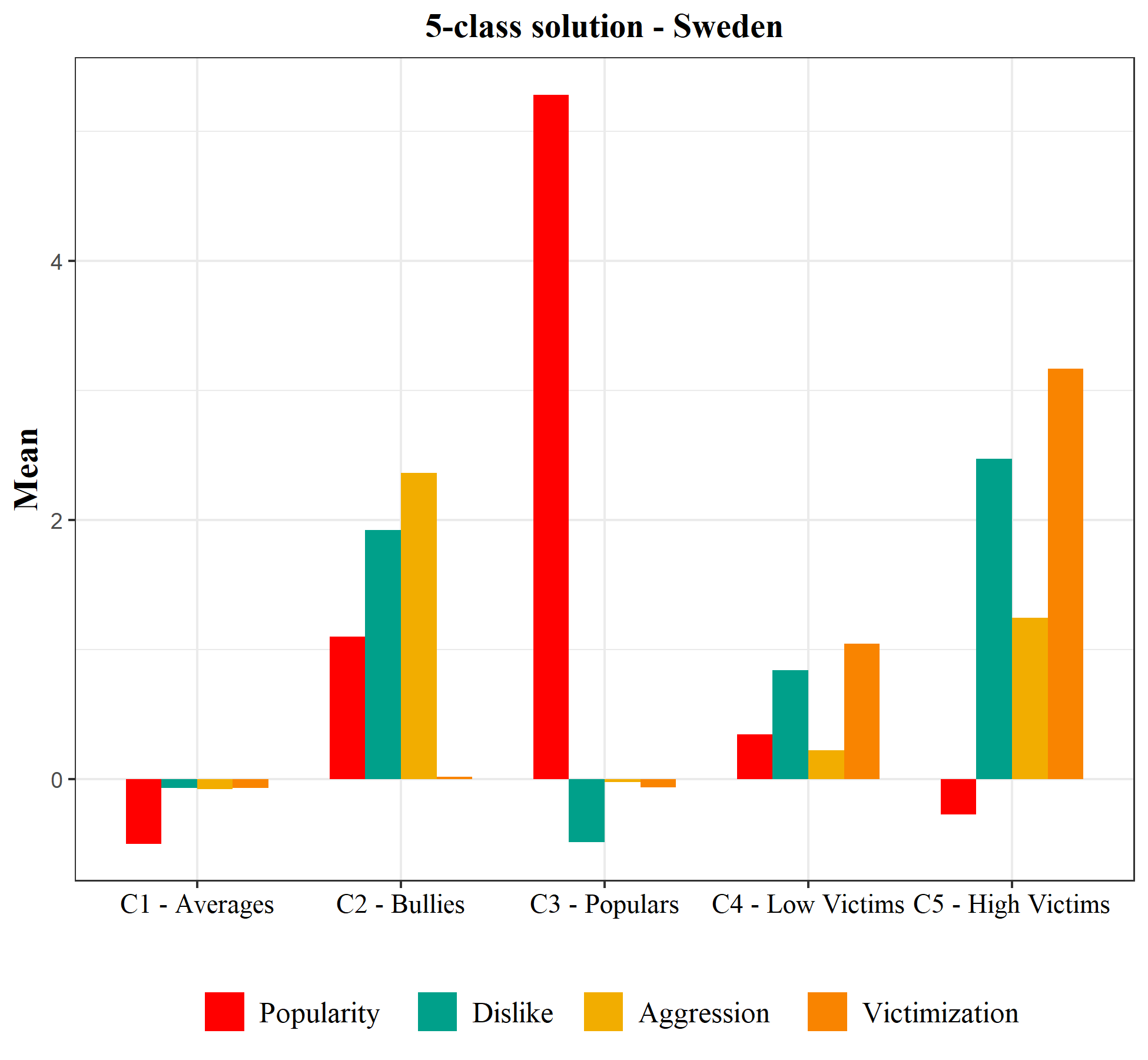

Supplement: Supplementary file 1 — Appendix S1 Supporting Information. [file SJOP-64-40-s001.docx]
